# Regulation of antiviral innate immune signaling and viral evasion following viral genome sensing

**DOI:** 10.1038/s12276-021-00691-y

**Published:** 2021-11-16

**Authors:** Kiramage Chathuranga, Asela Weerawardhana, Niranjan Dodantenna, Jong-Soo Lee

**Affiliations:** College of Veterinary Medicine, Chungnam National University, Daejeon, 34134 Korea

**Keywords:** Innate immunity, Post-translational modifications

## Abstract

A harmonized balance between positive and negative regulation of pattern recognition receptor (PRR)-initiated immune responses is required to achieve the most favorable outcome for the host. This balance is crucial because it must not only ensure activation of the first line of defense against viral infection but also prevent inappropriate immune activation, which results in autoimmune diseases. Recent studies have shown how signal transduction pathways initiated by PRRs are positively and negatively regulated by diverse modulators to maintain host immune homeostasis. However, viruses have developed strategies to subvert the host antiviral response and establish infection. Viruses have evolved numerous genes encoding immunomodulatory proteins that antagonize the host immune system. This review focuses on the current state of knowledge regarding key host factors that regulate innate immune signaling molecules upon viral infection and discusses evidence showing how specific viral proteins counteract antiviral responses via immunomodulatory strategies.

## Immunology: Viral infection and host defenses

In the initial stages of viral infection, the body relies on innate or non-specific immune signaling pathways to spur antiviral response mechanisms into action, however viruses have evolved to counteract these defense mechanisms. Jong-Soo Lee and colleagues from Chungnam National University in Daejeon, South Korea, review the signaling pathways and regulatory factors involved in these two opposing processes. The researchers detail the different molecular players involved in recognizing DNA or RNA from viruses and highlight the immune-modulating mechanisms by which viruses evade detection. A more complete knowledge of these pathways and their interactions could help drug developers identify new ways of treating or preventing viral infections. It could also provide insights into how autoimmunity and other problems can arise when these antiviral surveillance mechanisms fail to work correctly.

## Introduction

Viruses need to hijack the host cell machinery to replicate effectively; however, they must first overcome the host’s defenses. The efficacy of a viral infection depends on the comparative potency of the effector molecules used by the virus and the host. A critical determinant of whether a host succumbs to or can subvert a viral infection is the speed at which the host’s defenses are activated^1^. Almost all innate immune responses require an extended sequence of actions: pathogen sensing, signal transduction, transcription, translation, protein folding, and transport to the site of action. To initiate signaling upon viral infection, host cells detect viral DNA or RNA using a set of PRRs; these include retinoic acid-inducible gene-I (RIG-I)-like receptors (RLRs), toll-like receptors (TLRs), nucleotide-binding oligomerization domain (NOD)-like receptors (RNA sensors), cyclic GMP-AMP (cGAMP) synthase (cGAS), interferon gamma inducible protein 16 (IFI16), absent in melanoma 2 (AIM2), and dead-box helicase 41 (DDX41) (DNA sensors)^2,3^.

**Fig. 1:**
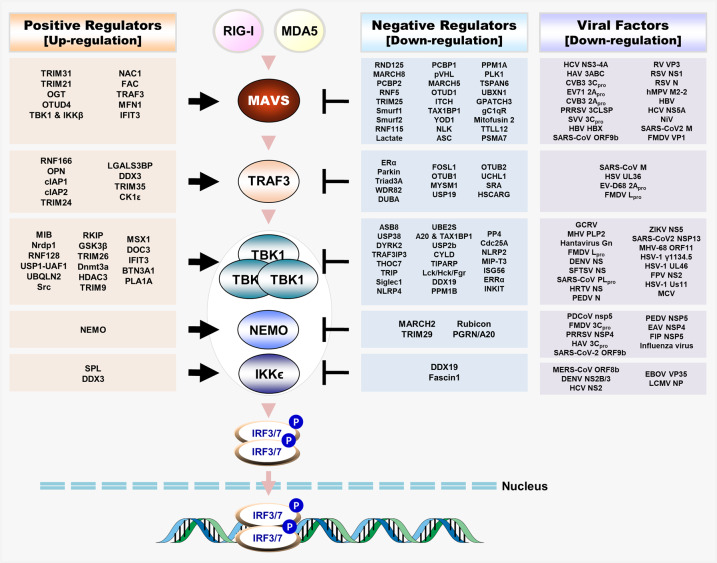
Regulatory host factors and interacting viral proteins of the RLR-mediated antiviral signaling pathway. Schematic representation of positive and negative regulatory host factors of Mitochondrial antiviral signaling protein (MAVS), TNF receptor-associated factor (TRAF3), TANK-binding kinase 1 (TBK1), NF-kappa-B essential modulator (NEMO), and IĸB kinase-ε (IKKε) through posttranslational modifications (PTMs) or other regulatory mechanisms and viral proteins interacting with MAVS, TRAF3, TBK1, NEMO, or IKKε for viral evasion of the host immune response. The RLR-MAVS pathway consists of RIG-I and MDA5 as the main viral RNA sensors and the downstream signaling molecules MAVS and TRAF3, which activate IRF3/IRF7 via the kinases IKK and TBK1/IKKε. (Note: Host factors and viral proteins involved in TBK1 regulation upon infection with both RNA and DNA viruses are indicated as being common regulators in the figure.).

**Fig. 2:**
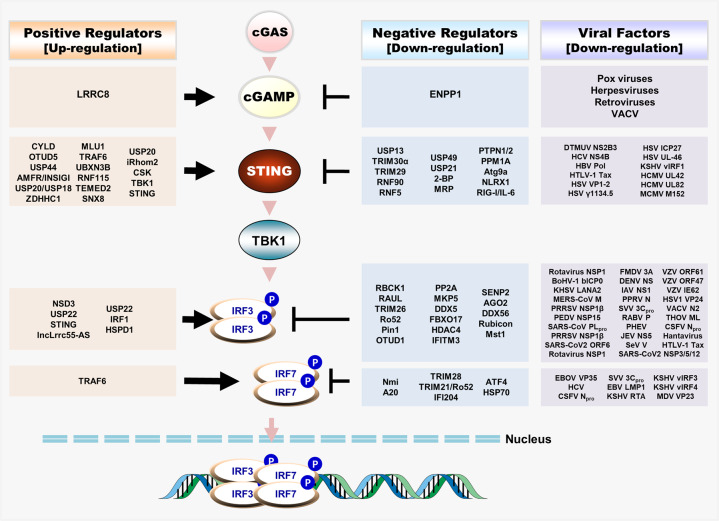
Regulatory host factors and interacting viral proteins of the cGAS-mediated antiviral signaling pathway. Schematic representation of positive and negative regulatory host factors of 2’,3’-cyclic GMP-AMP (2’,3’-cGAMP), stimulator of interferon gene (STING), Interferon regulatory factor 3 (IRF3), and IRF7 through posttranslational modifications (PTMs) or other modifications and viral proteins interacting with cGAMP or STING for viral evasion of the host immune response. The STING-mediated signaling pathway includes cGAS as the key sensor molecule that is mainly involved in the recognition of viral DNA. This recognition triggers cGAMP production and binding of cGAMP with STING, which leads to activation of IRF3/IRF7 and induction of type 1 IFNs. TBK1, IRF3, and IRF7 are involved in the IFN signaling cascade initiated upon sensing of RNA and DNA viruses. (Note: Host factors and viral proteins involved in IRF3/IRF7 regulation upon infection with both RNA and DNA viruses are indicated as being common regulators in the figure.).

Recognition of viral nucleic acids by PRRs triggers transduction of downstream signals mainly via adaptor proteins such as mitochondrial antiviral signaling protein (MAVS) or stimulator of interferon genes (STING), which induce expression of interferon (IFN)-stimulated genes via autocrine or paracrine mechanisms; the products of genes (proinflammatory cytokines, chemokines, and IFNs) inhibit viral replication and spread and induce activation of adaptive immune responses^4,5^. These antiviral signaling pathways play a crucial role in achieving an optimal outcome for the host; therefore, much attention has been devoted to identifying and understanding the signaling pathways and regulatory factors involved in antiviral innate immunity^6^ (Figs. 1, 2).

Conventional posttranslational modifications such as polyubiquitination and phosphorylation, unconventional posttranslational modifications such as acetylation and methylation, and other regulatory mechanisms such as physical interactions and translocations affect the production of IFN-β and inflammatory cytokines by targeting innate immune sensors and downstream signaling molecules (e.g., receptors, adaptors, enzymes, and transcription factors)^7,8^. These aforementioned modifications play a critical role in regulating the production of IFNs and inflammatory cytokines, which can, if production is unchecked, have deleterious effects on the host by promoting the development of autoimmune disorders, allergies, and other immunopathologies, as well as by activating and regulating the cellular status to exacerbate the severity of viral disease^9^.

It is not surprising that viruses exploit numerous strategies to enhance their replication. To establish efficient, lifelong infection and to initiate viral pathogenesis, a large portion of the viral genome encode numerous immunomodulatory proteins; the function of these proteins is to evade/disrupt the host immune system and ensure viral persistence^10^. From the perspective of the virus, these actions are critically important because viruses depend on living cells for replication. This review focuses on current knowledge regarding two factors. First, we summarize the posttranslational modifications (PTMs) and other regulatory mechanisms of signaling molecules downstream of the RNA/DNA sensing cascade that regulate efficient IFN responses and/or maintenance of host immune homeostasis. Second, we summarize how RNA/DNA viruses evade transduced host innate immune signals, which are initiated by PRRs, to establish a permissive state in host cells.

## Role of PTMs in regulating signal transduction

PTMs play an important role in regulating the stability, activity, subcellular localization, and folding of proteins. Advances in experimental techniques used to map and quantify PTMs have led to marked progress in these areas. Such techniques have identified a number of PTMs that alter the innate immune response by regulating protein function, abundance, catalysis, interactions, or subcellular localization without necessarily requiring induction of a new transcriptional program^8,11^. Additionally, some of these PTMs are highly dynamic and fully reversible, allowing both initiation and resolution of responses. Phosphorylation, a process by which a phosphoryl group is attached to a serine, threonine, tyrosine, histidine, or aspartate residue, is a well-studied PTM regulated by the opposing actions of protein kinases and phosphatases; this PTM plays an important role in innate immunity^11,12^. The introduction of a phosphoryl group imparts a negative (–2) charge at physiological pH, resulting in a major biophysical perturbation of protein structure. This is manifested by conformational changes that alter enzymatic activity and/or protein–protein interactions^13^. Ubiquitination is another important PTM. During ubiquitination, proteins are modified via covalent attachment of a small 76-amino acid protein called ubiquitin, which (as the name implies) is expressed ubiquitously and is highly conserved in all eukaryotes^14^. Ubiquitination is inversely regulated by ubiquitin activating (E1), ubiquitin-conjugating (E2), and ubiquitin protein ligase (E3) enzymes and by deubiquitinating enzymes (DUBs); thus, it plays a critical role in regulating innate immune signal transduction. In contrast to phosphorylation, a single target site can be modified by a single ubiquitin molecule (monoubiquitination) or by chains of linked ubiquitin molecules (polyubiquitination)^15^. Ubiquitin chains can be classified topologically into one of four types according to architecture: homogeneous chains, multiple chains (in which one substrate is separately modified by distinct chains), mixed chains (in which a tandem chain contains two linkage types), and branched chains^16,17^. Lysine 48 (K48)-linked polyubiquitination induces proteasomal degradation of the target protein, whereas K63-linked polyubiquitination mediates signal transduction^16,17^. Monoubiquitination, linear polyubiquitination, and K6-, K11-, K27-, K29-, and K33-linked ubiquitination are being investigated intensely to determine their divergent roles in innate immunity^15^. Similar to conventional PTMs, unconventional PTMs also play a role in innate immune signal transduction^8^. The transfer of acetyl groups from acetyl coenzyme A (acetyl-CoA) to the ε amino acid groups of lysine residues (a process termed acetylation) results in charge neutralization, which alters the biological properties of proteins; in addition, lysine and arginine residues are inversely regulated by methyltransferases (a process termed methylation) and demethylases, and both acetylation and methylation play important roles in innate immune signaling^18^. Below we summarize the PTMs and other regulatory mechanisms of signaling molecules downstream of the RNA/DNA sensing cascade (also see Tables 1, 2, and 3).

**Table 1:** Host regulators of RLR-initiated antiviral signaling.

Signaling molecule	Classification		Regulator	Function	Ref.
MAVS	PTMs	Positive	MAVS	Aggregation	^45^
			TRIM31	Aggregation	^47^
			TRIM21	K27-linked ubiquitination	^49^
			OGT	K63-linked ubiquitination	^48^
			OTUD4	Deubiquitination	^51^
			TBK1 and IKKβ	Recruitment of IRF3 for its phosphorylation by TBK1	^12^
		Negative	RNF125	Ubiquitination	^68^
			MARCH8	K27-linked ubiquitination	^69^
			PCBP2	K48-linked ubiquitination	^65^
			RNF5	K48-linked ubiquitination	^52^
			TRIM25	K48-linked ubiquitination	^59^
			Smurf1	K48-linked ubiquitination	^60^
			Smurf2	K48-linked ubiquitination	^61^
			RNF115	K48-linked ubiquitination	^58^
			PCBP1	K48-linked ubiquitination	^50^
			pVHL	K48-linked ubiquitination	^62^
			MARCH5	K48-linked ubiquitination	^63^
			OTUD1	K48-linked ubiquitination	^67^
			ITCH	K48-linked ubiquitination	^66^
			TAX1BP1	K48-linked ubiquitination	^66^
			YOD1	Deubiquitination	^64^
			NLK	Phosphorylation and degradation	^70^
			PPM1A	Dephosphorylation	^71^
	Other regulatory mechanisms	Positive	MFN1	Abrogation of virus-induced redistribution of MAVS	^55^
			IFIT3	Induction of bridging between MAVS and TBK1	^168^
			NAC1	Induction of bridging between MAVS and TBK1	^56^
			FAK	Activation	^57^
			TRAF3	Activation	^54^
		Negative	PLK1	Disruption of the MAVS-TRAF3 interaction	^77^
			UBXN1	Interference with MAVS oligomerization and disruption of the MAVS/TRAF3/TRAF6 signalosome	^74^
			GPATCH3	Disruption of virus-induced MAVS signalosome formation	^76^
			gC1qR	Physical interaction	^79^
			Mitofusin 2	Physical interaction	^80^
			TTLL12	Direct interaction with MAVS, TBK1 and IKKε; inhibition of the interactions of MAVS with other signaling molecules	^73^
			Lactate	Direct interaction with MAVS to prevent MAVS aggregation	^72^
			ASC	Physical interaction	^81^
			PSMA7	Physical interaction	^82^
			Rac1	Inhibition of MAVS ubiquitination, aggregation, and activation	^78^
			LGP2	Inhibition of IKKε binding	^75^
TRAF3	PTMs	Positive	RNF166	Ubiquitination	^115^
			OPN	Deubiquitination	^117^
			cIAP1	K63-linked ubiquitination	^111^
			cIAP2	K63-linked ubiquitination	^111^
			TRIM24	K63-linked ubiquitination	^113^
			LGALS3BP	K63-linked ubiquitination	^112^
			DDX3	K63-linked ubiquitination	^110^
			TRIM35	K63-linked ubiquitination	^114^
			CK1ε	Phosphorylation and promotes K63-linked ubiquitination	^116^
		Negative	ERα	K48-linked ubiquitination	^128^
			Parkin	K48-linked ubiquitination	^130^
			Triad3A	K48-linked ubiquitination	^131^
			WDR82	K48-linked ubiquitination	^129^
			DUBA	Deubiquitination	^121^
			MYSM1	Deubiquitination	^120^
			USP19	Deubiquitination	^122^
			FOSL1	Deubiquitination	^125^
			OTUB1	Deubiquitination	^123^
			OTUB2	Deubiquitination	^123^
			UCHL1	Deubiquitination	^124^
			SRA	Deubiquitination	^127^
			HSCARG	Deubiquitination	^126^
	Other regulatory mechanisms	Positive	DOK3	TRAF3/TBK1 complex formation	^118^
			RAB1B	Facilitation of the interaction with MAVS	^119^
		Negative	NEMO	Disruption of the MAVS-TRAF3 complex	^132^
NEMO	PTMs	Positive	NEMO	K27-linked ubiquitination of TRIM23	^139^
		Negative	MARCH2	K48-linked ubiquitination	^140^
			TRIM29	K48-linked ubiquitination	^141^
			Rubicon	Inhibition of ubiquitination	^142^
			PGRN/A20	Deubiquitination	^143^
IKKε	PTMs	Negative	DDX19	Degradation	^210^
	Other regulatory mechanisms	Positive	SPL	Physical interaction	^208^
			DDX3	Activation	^209^
		Negative	Fascin1	Physical interaction	^211^

**Table 2:** Host regulators of cGAS-initiated antiviral signaling.

Signaling molecule	Classification		Regulator	Function	Ref.
2′,3′-cGAMP	Positive		LRRC8	Transportation	^290^
	Negative		ENPP1	Hydrolysis	^291^
				Physical interaction & hydrolysis	^292^
STING	PTMs	Positive	AMFR/INSIG1	K27-linked ubiquitination	^304^
			MUL1	K63-linked ubiquitination	^301^
			TRAF6	K63-linked ubiquitination	^302^
			UBXN3B	K63-linked ubiquitination	^303^
			RNF115	K63-linked ubiquitination	^59^
			CYLD	Deubiquitination	^308^
			OTUD5	Deubiquitination	^309^
			USP44	Deubiquitination	^307^
			USP20/USP18	Deubiquitination	^305^
			USP20	Deubiquitination	^306^
			iRhom2	Deubiquitination	^310^
			CSK	Phosphorylation	^315^
			TBK1	Phosphorylation	^313^
			STING	Palmitoylation	^312^
		Negative	USP13	K33-linked ubiquitination	^324^
			TRIM30α	K48-linked ubiquitination	^321^
			TRIM29	K48-linked ubiquitination	^320^
			RNF90	K48-linked ubiquitination	^319^
			RNF5	K48-linked ubiquitination	^318^
			USP49	Deubiquitination	^323^
			USP21	Deubiquitination	^322^
			PTPN1/2	Dephosphorylation & degradation	^325^
			PPM1A	Dephosphorylation	^314^
			2-BP	Inhibition of palmitoylation	^311,312^
	Other regulatory mechanisms	Positive	ZDHHC1	Physical interaction	^315^
			TMED2	Physical interaction	^316^
			SNX8	Translocation	^317^
		Negative	Atg9a	Colocalization	^329^
			MRP	Physical interaction	^326^
			NLRX1	Physical interaction	^327^
			RIG-1/IL-6	Degradation	^328^

**Table 3:** Host regulators commonly involved in RLR/cGAS-initiated antiviral signaling.

Signaling molecule	Classification		Regulator	Function	Ref.
TBK1	PTMs	Positive	MIB	K63-linked ubiquitination	^161^
			TBK1	K63-linked ubiquitination	^154^
			Nrdp1	K63-linked ubiquitination	^163^
			RNF128	K63-linked ubiquitination	^162^
			USP1–UAF1 complex	Deubiquitination	^164^
			UBQLN2	Phosphorylation	^159^
			Src	Autophosphorylation	^160^
			TBK1	Autophosphorylation	^155^
			RKIP	Autophosphorylation	^158^
			GSK3β	Self-association and autophosphorylation	^157^
			Dnmt3a	Recruitment of HDAC9 for deacetylation	^165^
			HDAC3	Deacetylation	^166^
			TRIM9	Recruitment of GSK3β for activation	^157^
		Negative	ASB8	K48-linked ubiquitination	^172^
			USP38	K48-linked ubiquitination	^176^
			DYRK2	K48-linked ubiquitination	^174^
			THOC7	K48-linked ubiquitination	^175^
			TRIP	K48-linked ubiquitination	^173^
			Siglec1	Recruitment of TRIM27 for K48-linked ubiquitination	^177^
			NLRP4	Recruitment of DTX4 for K48-linked ubiquitination of TBK1	^178^
			A20 and TAX1BP1	Inhibition of K63-linked ubiquitination	^179^
			UBE2S	Recruitment of USP15 for deubiquitination	^181^
			USP2b	Deubiquitination	^180^
			CYLD	Deubiquitination	^37^
			TIPARP	ADP-ribosylation & TBK1 deactivation	^183^
			Lck/Hck/Fgr	Disruption of dimerization and activation	^182^
			PPM1B	Dephosphorylation	^185^
			PP4	Dephosphorylation and Deactivation	^186^
			Cdc25A	Dephosphorylation	^184^
	Other regulatory mechanisms	Positive	MSX1	Induction of the assembly of TBK1-associated complexes	^118^
			DOK3	Facilitation of TRAF3/TBK1 complex formation	^170^
			IFIT3	Bridging of TBK1 to MAVS on mitochondria	^168^
			BTN3A1	Transport of the TBK1/IRF3 complex to the perinuclear region	^167^
			PLA1A	Phosphorylation and modulation of mitochondrial morphology	^171^
			TRIM26	Induction of TBK1/NEMO interaction	^169^
		Negative	NLRP2	Disruption of IRF3 binding	^187^
			MIP-T3	Inhibition of TRAF3/TBK1 complex formation	^189^
			ISG56	Disruption of the interaction between MITA and MAVS or TBK1	^190^
			ERRα	Inhibition of the TBK1-IRF3 interaction	^188^
			INKIT	Physical interaction	^191^
IRF3	PTMs	Positive	NSD3	Methylation	^223^
			HSPD1	Phosphorylation and dimerization	^61^
			lncLrrc55-AS	Phosphorylation	^221^
		Negative	RBCK1	Ubiquitination	^226^
			RAUL	K48-linked ubiquitination	^229^
			TRIM26	K48-linked ubiquitination	^230^
			Ro52	Ubiquitination	^227^
			Pin1	Ubiquitination	^225^
			OTUD1	Deubiquitination	^231^
			Mst1	Phosphorylation	^235^
			PP2A	Dephosphorylation	^234^
			MKP5	Dephosphorylation	^233^
			DDX5	Dephosphorylation	^237^
			FBXO17	Dephosphorylation	^236^
			HDAC4	Inhibition of phosphorylation	^238^
			IFITM3	Autophagic degradation	^232^
			SENP2	DeSUMOylation	^239^
	Other regulatory mechanisms	Positive	USP22	Nuclear translocation	^224^
			IRF1	Activation	^222^
		Negative	AGO2	Inhibition of the IRF3–CBP interaction	^242^
			DDX56	Inhibition of nuclear translocation	^240^
			Rubicon	Inhibition of dimerization	^241^
IRF7	PTMs	Positive	TRAF6	K63-linked ubiquitination	^269^
		Negative	Nmi	K48-linked ubiquitination	^270^
			A20	Deubiquitination	^274^
			TRIM28	SUMOylation	^272^
			TRIM21/Ro52	Degradation	^271^
	Other regulatory mechanisms	Negative	IFI204	Physical interaction	^275^
			ATF4	Physical interaction	^276^
			HSP70	Physical interaction	^277^

## Innate immune evasion strategies used by RNA and DNA viruses

Viruses that have evolved with their host develop strategies to evade the innate immune system and ensure their replication and survival. Individual viruses or virus families use different strategies. This review explores the different mechanisms used by RNA and DNA viruses to subvert the functions of individual signaling molecules in the type 1 interferon (IFN) pathway. Many viruses use proteases to cleave target proteins^19^, while some viral proteins promote the degradation of target innate immune signaling molecules^20,21^. Furthermore, viral deubiquitinase enzymes remove K63-linked polyubiquitin chains from signaling molecules to prevent their activation^22,23^, and viral E3 ubiquitin ligases transfer K48-linked polyubiquitin moieties to target molecules to trigger their proteasomal degradation^24^. Some viral proteins recruit host E3 ubiquitin ligases to polyubiquitinate signaling molecules and increase their proteasomal degradation^25^. The formation of signaling molecule complexes is crucial for downstream transduction of innate immune signals. Direct interactions with viral proteins inhibit the formation of signaling complexes such as the TRAF3, TANK, and TBK1 complexes^26,27^. Another important mechanism of immune evasion is physical interaction between viral proteins and host signaling molecules, which prevents activation, dimerization, phosphorylation, or nuclear translocation^28,29^. Below, we summarize the mechanisms underlying innate immune evasion mediated by viral proteins (also see Tables 4 and 5).

**Table 4:** Viral evasion mechanisms of RLR-initiated antiviral signaling.

Signaling molecules	Virus	Virulence factor	Function	Ref.
MAVS	HCV	NS3-4A	Cleavage	^38,89^
	HAV	3ABC	Cleavage	^91^
	CVB3	3C_pro_	Cleavage	^85^
	EV71	2A_pro_	Cleavage	^83^
	CVB3	2A_pro_	Cleavage	^87^
	PRRSV	3CLSP	Cleavage	^88^
	SVV	3C_pro_	Cleavage	^86^
	HBV	HBX	Ubiquitination	^95^
	RV	NSP1	Degradation	^93^
	SARS-CoV	ORF9b	Degradation	^97^
	SARS-CoV-2	M	Inhibition of RIG-I, MAVS, TRAF3 and TBK-1 complex formation	^26^
	SARS-CoV-2	M	Inhibition of MAVS aggregation	^105^
	RV	VP3	Proteosomal degradation	^94^
	RSV	NS1	Inhibition of the MAVS-RIG-I interaction	^102^
	RSV	N	Localization with MAVS in inclusion bodies	^103^
	hMPV	M2-2	Inhibition of TRAF3-, TRAF5- and TRAF6-mediated recruitment of MAVS	^104^
	HBV		Recruitment of LUBAC & disruption of MAVS signalosome formation	^96^
	HCV		Recruitment of PCBP2 to MAVS and induction of K48-linked ubiquitination	^25^
	HCV		Regulation of the interaction between GP73 and MAVS for proteasomal degradation	^98^
	NiV	V	Stabilization of UBXN1 and enhancement of its interaction with MAVS	^100^
	HCV	NS5A	Inhibition of the MAVS-TRAF3 interaction	^99^
	FMDV	VP1	Inhibition of the TRAF3-MAVS interaction	^101^
TRAF3	SARS-CoV	M	Inhibition of TRAF3, TANK, and TBK1/IKKε complex formation	^27^
	FMDV	Lb_pro_	Deubiquitination	^133^
	HSV	UL36	Deubiquitination	^134^
	EV-D68	2A_pro_	Cleavage	^135^
NEMO	PDCoV	nsp5	Cleavage	^150^
	FMDV	3C_pro_	Cleavage	^144^
	PRRSV	NSP4	Cleavage	^146,147^
	HAV	3C_pro_	Cleavage	^145^
	PEDV	NSP5	Cleavage	^149^
	EAV	NSP4	Cleavage	^147^
	FIP	NSP5	Cleavage	^148^
	Influenza virus	–	Enhancement of the PGRN level to inhibit K63-linked ubiquitination	^143^
	SARS-CoV-2	ORF9b	Deubiquitination of NEMO	^151^
TBK1	GCRV	–	K48-linked ubiquitination	^24^
	MHV	PLP2	Deubiquitination	^195^
	FMDV	L_pro_	Inhibition of TBK1 ubiquitination and activation	^133^
	SFTSV	NS	Sequestration of the TBK1/IKKe complex into inclusion bodies	^197,198^
	SARS-CoV	PLpro	Disruption of the STING-TRAF3-TBK1 interaction	^204^
	DENV	NS	Inhibition of phosphorylation	^194^
	HRTV	NS	Inhibition of TBK1 and IRF3 interaction	^201^
	PEDV	N	Inhibition of the association between TBK1 and IRF3 by sequestration	^200^
	MCV	MC159/MC160	Impairment of activation	^205^
	ZIKV	NS5	Impairment of activation	^202^
	SARS-CoV-2	NSP13	Inhibition of phosphorylation	^192^
	SARS-CoV-2	NSP13	Disruption of the TBK1-MAVS interaction	^203^
	HRTV	NS	Inhibition of phosphorylation	^193^
IKKε	MERS-CoV	ORF8b	Inhibition of HSP70-dependent activation	^213^
	DENV	NS2B/3	Binding and inhibition of kinase activity	^212^
	HCV	NS2	Inhibition of IRF3 phosphorylation via interaction with IKKε	^216^
	EBOV	VP35	Impairment of the IKKε–IRF3, IKKε–IRF7, and IKKε–IPS-1 interactions	^214^
	LCMV	NP	Inhibition of catalytic activity	^215^
IRF3	PRRSV	NSP1β	Inhibition of activation	^228^
	RABV	P	Inhibition of activation	^245^
	PHEV	–	Blockade of activation	^244^
	JEV	–	Inhibition of nuclear translocation	^254^
	SARS-CoV-2	ORF6	Inhibition of nuclear translocation	^192^
	SARS-CoV-2	ORF6	Inhibition of activation	^249^
	SARS-CoV-2	NSP12	Inhibition of nuclear translocation	^250^
	SARS-CoV-2	NSP5	Inhibition of nuclear translocation	^251^
	SARS-CoV-2	NSP3	Cleavage	^252^
	JEV	NS5	Inhibition of nuclear translocation	^253^
	SeV	V	Inhibition of nuclear translocation	^255^
	Rotavirus	NSP1	Blockade of dimerization	^264^
	THOV	ML	Blockade of dimerization	^263^
	CSFV	N_pro_	Proteosomal degradation	^257^
	Hantavirus	–	Inhibition of phosphorylation	^260^
	HTLV-1	Tax	Inhibition of phosphorylation	^261^
	FMDV	3A	Inhibition of phosphorylation	^259^
	DENV	NS	Inhibition of phosphorylation	^194^
	MERS-CoV	M	Inhibition of phosphorylation	^262^
	PEDV	NSP15	Inhibition of activation	^248^
	IAV	NS1	Inhibition of activation	^243^
	PPRV	N	Inhibition of activation	^247^
	SARS-CoV	PLpro	Inhibition of activation	^246^
	SVV	3C_pro_	Degradation	^256^
	Rotavirus	NSP1	Degradation	^258^
IRF7	EBOV	VP35	Enhancement of PIAS1-mediated SUMOylation	^278^
	HCV	–	Inhibition of nuclear translocation	^279^
	CSFV	N_pro_	Physical interaction	^280^
	SVV	3C_pro_	Degradation	^256^

**Table 5:** Viral evasion mechanisms of cGAS-initiated antiviral signaling.

Signaling molecules	Virus	Virulence factor	Function	Ref
cGAMP	Poxviruses, Herpesviruses, Retroviruses	–	Transport	^293,294^
	VACV	Poxin (B2R)	Cleavage	^295^
STING	DTMUV	NS2B3	Cleavage	^343^
	HCV	NS4B	Cleavage	^335^
	HBV	Pol	K63-linked ubiquitination	^338^
	HSV-1	VP1-2	Deubiquitination	^334^
	HTLV-1	Tax	Deubiquitination	^22^
	HCMV	IE86	Proteosomal degradation	^340,341^
	HSV-1	γ_1_134.5	Physical interaction	^332^
	HSV-1	ICP27	Physical interaction	^331^
	HSV-1	UL-46	Physical interaction	^333^
	KSHV	vIRF1	Physical interaction	^336^
	HCMV	UL42	Physical interaction	^342^
	HCMV	UL82	Physical interaction	^339^
	MCMV	M152	Physical interaction	^337^
TBK1	MHV-68	ORF11	Physical interaction	^345^
	HSV-1	γ_1_1–34.5	Physical interaction	^344^
	HSV-1	UL46	Physical interaction	^333^
	FPV	NS2	Physical interaction	^346^
	HSV-1	Us11	Degradation	^196^
IRF3	BoHV-1	bICP0	Proteosomal degradation	^348^
	VZV	ORF61	Proteosomal degradation	^351^
	KHSV	LANA2 (vIRF3)	Physical interaction	^29^
	HSV-1	VP24	Physical interaction	^347^
	VZV	ORF47	Physical interaction	^350^
	VZV	IE62	Inhibition of phosphorylation	^349^
	VACV	N2	Inhibition of nuclear translocation	^351^
IRF7	EBV	LMP1	K63-linked ubiquitination	^352^
	KSHV	RTA	Degradation	^354^
	KSHV	vIRF4	Physical interaction	^356^
	MDV	VP23	Physical interaction	^353^
	KHSV	vIRF3	Physical interaction	^355^
	KSHV	LANA2 (vIRF3)	Physical interaction	^29^

## RNA-induced signal transduction and mechanisms underlying viral evasion of host immunity

RLR (RIG-I-like receptor) family receptors are the main PRRs that detect intracellular viral RNA^30,31^. The RLR family comprises RIG-I, melanoma differentiation-associated gene 5 (MDA5), and laboratory of genetics and physiology 2 (LGP2). RIG-I and MDA5 are typical PRRs, whereas LGP2 is a regulator of RIG-I and MDA5 mediated signal transduction^32,33^. RIG-I and MDA5 contain two N-terminal caspase-recruitment domains^34^, a central DExD/H-box helicase domain, and a C-terminal domain (CTD). RIG-I and MDA5 bind to viral RNA in the cytoplasm via an RNA binding motif^30,31^, after which the signaling domain interacts with the downstream adaptor molecule MAVS via a CARD-CARD-mediated interaction. This interaction causes aggregation of MAVS to form a prion-like protein complex, which relays the signal to kinases such as TANK-binding kinase 1 (TBK-1) and IĸB kinase-ε (IKKε). Activation of this cascade results in phosphorylation of the transcription factors IFN-regulating factor (IRF)-3 and IRF-7^31,35,36^. Finally, nuclear translocation of IRF-3 and IRF-7 induces the expression of type 1 IFN genes and other antiviral genes^37^. However, RNA viruses employ strategies to evade these RLR-mediated innate immune responses. Below, we describe the activation and regulatory mechanisms of the major innate signaling molecules, along with the immunomodulatory mechanisms by which viruses evade them.

## Regulation of MAVS by host factors

MAVS, also called IPS1, VISA, and CARDIF, is a key adaptor protein for RIG-I-like receptor-initiated signal transduction. Upon viral infection, RIG-I and MDA5 bind to MAVS, thereby activating downstream signal transduction. The MAVS protein, which contains 540 amino acids encoded by the nuclear genome^38^, is localized predominantly on the mitochondrial outer membrane. However, experimental evidence shows that it also localizes to mitochondrial-associated endoplasmic reticulum membranes and peroxisomes^39–41^. MAVS contains three domains: a CARD, a middle proline-rich region, and a C-terminal transmembrane^42^ domain. The CARD interacts with CARDs in RIG-I and MDA5, activating MAVS, whereas the proline-rich region interacts with the tumor necrosis factor receptor-related factor (TRAF) family members TRAF2, TRAF3, TRAF5, and TRAF6 to activate downstream signaling^43^. The TM domain plays a crucial role by ensuring the localization of MAVS to the mitochondrial outer membrane^44^. Upon binding to the CARDs of RIG-I and MDA5, MAVS rapidly forms prion-like aggregates, which convert other MAVS proteins present on the mitochondrial outer membrane into prion-like aggregates^45^. Activation of MAVS through aggregation recruits TRAF2, TRAF3, TRAF5, and TRAF6 via the PRR to promote the formation of the TBK1 complex (comprising TBK1, IĸB kinase, IKKε, and NEMO)^46^. It is not surprising that the expression of MAVS is regulated to ensure that RLR-mediated signaling cascades are not activated rapidly upon stimulation; indeed, its function at this stage of viral infection is to prevent rapid viral replication.

Self-association and prion-like aggregate formation are markers of MAVS activation^45^. The E3 ubiquitin ligase Tripartite motif-containing protein (TRIM) 31 interacts with MAVS and catalyzes K63-linked polyubiquitination of aa residues K10, K311, and K461 in MAVS to promote the formation of aggregates. Interestingly, this phenomenon occurs upon viral infection in the presence of RIG-I; thus, recruitment of RIG-I may be required for TRIM31-mediated MAVS aggregation upon viral infection^47^. Moreover, K63-linked polyubiquitination is enhanced by O-linked N-acetyl glucosamine (O-GlcNAc) transferase (OGT)-mediated glycosylation of MAVS^48^. Another recent study suggested that K27-linked polyubiquitination of K325 in MAVS by the E3 ubiquitin ligase TRIM21 promotes downstream signaling activation. The PRY-SPRY domain of TRIM21 interacts with MAVS, while the RING (Really Interesting New Gene) domain transfers the E3 ubiquitin protein complex to MAVS, resulting in recruitment of TBK1 to MAVS^49^. K48-linked ubiquitination of MAVS leads to its proteasomal degradation^50^; thus, proteins that inhibit MAVS K48-linked ubiquitination are positive regulators of MAVS-mediated signaling. Ovarian tumor family deubiquitinase 4 (OTUD4) removes K48-linked ubiquitin chains from MAVS to inhibit its degradation^51^. Moreover, the expression of cyclophilin A is upregulated upon viral infection; cyclophilin A competes with TRIM25 for binding to MAVS. Inhibiting TRIM25 promotes MAVS ubiquitination and degradation^52,53^.

Phosphorylation is an important PTM that regulates MAVS signaling. Activated MAVS recruits TBK1 and IKKε to the complex. These kinases mediate the phosphorylation of MAVS, enabling the recruitment of IRF3. Recruited IRF3 is phosphorylated by TBK1, which increases its homodimerization and nuclear translocation. Similar to PTMs, non-PTMs play a crucial role in regulating MAVS signaling^12^. Importantly, TRAF3 interacts with MAVS (aa 450–468), resulting in activation of MAVS signaling^54^. Mitofusin 1 (MFN1) binds to MAVS to increase MAVS redistribution; MFN1 positively regulates the RLR-mediated innate antiviral response^55^. Furthermore, nucleus accumbens-associated 1 (NAC1), a member of the BTB/POZ family, acts as a bridge between MAVS and TBK1, thereby activating downstream signaling^56^. In addition, focal adhesion kinase (FAK) interacts with MAVS at the mitochondrial membrane in a viral infection-dependent manner to potentiate MAVS-mediated signaling via a kinase-independent mechanism^57^.

Negative regulation of MAVS is mediated mainly by K48-linked ubiquitination of MAVS, signaling blockade, autophagy, and apoptosis. K48-linked polyubiquitination of MAVS triggers its proteasomal degradation and abrogates RLR-mediated signal transduction. Experimental evidence has shown that several E3 ubiquitin ligases are involved in K48-linked ubiquitination of MAVS and its proteasomal degradation; these ligases include Ring finger protein 5 (RNF5), RNF115, TRIM25, Smurf1, Smurf2, von Hippel-Lindau protein (pVHL), and membrane-associated RING finger protein 5 (MARCH5)^52,58–63^. Importantly, the ubiquitin thioesterase OTU1 (YOD1) cleaves the K63-linked ubiquitin moiety and abrogates the formation of prion-like aggregates by MAVS, thereby attenuating IRF3-mediated production of IFN-β^64^. Moreover, interactions between several proteins mediate MAVS ubiquitination and degradation via recruitment of E3 ubiquitin ligases. For example, poly(RC) binding protein 1/2 (PCBP1/PCBP2)- and tax1-binding protein 1 (TAX1PB1)-mediated K48-linked ubiquitination of MAVS via AIP4/ITCH triggers proteasomal degradation of MAVS^50,65,66^. Similarly, Smurf1-mediated K48-linked ubiquitination is upregulated by OTUD1^67^. The E3 ubiquitin ligase RNF125 conjugates ubiquitin to MAVS, thereby suppressing its function, and K27-linked ubiquitination of MAVS mediated by the E3 ubiquitin ligase MARCH8 recruits the autophagy protein NDP52, resulting in lysosomal degradation of MAVS^68,69^. Additional mechanisms that negatively regulate MAVS-mediated RLR signaling are phosphorylation and degradation of MAVS via Nemo-like kinase (NLK)^70^. Protein phosphatase magnesium-dependent 1A (PPM1A; also called PP2Cα) is an inherent component of the TBK1/IKKε complex, which targets both MAVS and TBK1/IKKε for dephosphorylation, thereby disrupting MAVS-driven formation of the signaling complex^71^.

Direct protein–protein interactions and signal blockade are other mechanisms that downregulate MAVS-mediated RLR signaling. Recent studies have shown that lactate, the end product of anaerobic glycolysis, acts as a negative regulator of RLR signal transduction by interacting with the TM domain of MAVS and preventing its mitochondrial localization and aggregation^72^. Tubulin tyrosine ligase-like protein 12 (TTLL12) interacts with MAVS, TBK1, and IKKε to prevent interactions between MAVS and other molecules. However, upon viral infection, TTLL12 expression decreases, thereby activating downstream MAVS signaling via the release MAVS blockade^73^. During the late stage of viral infection, MAVS function is negatively regulated by UBX-domain-containing protein 1 (UBXN1). The expression of UBXN1 increases at the late stage of infection, and it then competes with TRAF3/TRAF6 for binding to MAVS^74^. Similar to UBXN1, LGP2 binds to MAVS and prevents the interaction between MAVS and IKKε^75^. Additionally, gpatch domain-containing protein 3 (GPATCH3) binds to MAVS to prevent MAVS/TRAF6/TBK1 complex formation^76^, whereas binding of polo-like kinase 1 (PLK-1) to MAVS disrupts its interaction with TRAF3^77^. The Rho family small guanosine triphosphatase Ras-related C3 botulinum toxin substrate 1 (Rac1) limits the interaction between MAVS and the E3 ligase TRIM31, thereby inhibiting MAVS ubiquitination, aggregation, and activation^78^. Moreover, physical interactions between the gC1qR^79^, mitofusin^80^, ASC^81^, and PSMA7^82^ proteins and MAVS subvert MAVS function during viral infection.

## Regulation of MAVS by viral proteins

From the perspective of the virus, it is important to avoid the host innate immune response during the early stage of infection. Since MAVS plays a critical role as a central adaptor molecule in the RLR-mediated signaling cascade, the genomes of many viruses encode proteins that interfere with MAVS. For example, enterovirus 71 (EV71) cysteine protease 2A_pro_ cleaves MAVS at Gly209, Gly251, and Gly265^83^. This was the first viral protein found to cleave MAVS at multiple aa residues. The small RNA viruses human rhinovirus C, coxsackievirus B3 (CVB3), and Seneca Valley virus (SVV) encode a cysteine protease, 3C^pro^, which cleaves MAVS at Gln148 to prevent signal transduction^84–86^. In addition, CVB3 encodes another MAVS-cleaving protease named 2A_pro_; however, its cleavage site is unclear^87^. Porcine reproductive and respiratory syndrome virus (PRRSV) produces a 3C-like serine protease (3CLSP) that cleaves MAVS at Glu268^88^. Additionally, NS3-4A of hepatitis C virus (HCV)^38,89^ and the 3ABC precursor of 3C^90^ of hepatitis A virus^91^ cleave MAVS to disrupt activation of its downstream signaling^92^. The E3 ubiquitin ligase-like activity of rotavirus NSP1 means that its interaction with the MAVS CARD or TM domain leads to ubiquitin-dependent proteasomal degradation of MAVS^93^. Additionally, the structural protein VP3 of RV upregulates the phosphorylation of MAVS, leading to its K48-linked ubiquitination-mediated proteasomal degradation^94^. Hepatitis B virus (HBV) protein X (HBX) binds to MAVS and promotes its ubiquitination and proteasomal degradation via an unknown E3 ubiquitin ligase^95^. Additionally, HBV-induced Parkin recruits the linear ubiquitin assembly complex to mitochondria and abrogates IFN-β synthesis^96^. Severe acute respiratory syndrome coronavirus (SARS-CoV-2) open reading frame 9b (ORF-9b) catalyzes K48-linked ubiquitination of MAVS via the PCBP2-AIP4 axis^97^. Moreover, HCV infection induces the expression of Golgi protein 73 (GP73), which mediates the proteasomal degradation of MAVS^98^. HCV infection upregulates NLRX1 and recruits PCBP2 to MAVS, thereby triggering K48-linked ubiquitination and degradation of MAVS with the help of AIP4^25^. In addition, the interaction between the HCV NS5A protein and MAVS prevents the binding of the latter to TRAF3 and TRAF6^99^. The Nipah virus (NiV) V protein interacts directly with UBXN1 to enhance the interaction between MAVS and UBXN1 via protein stabilization^100^. A recent study showed that the wild-type VP1 (83E) but not the mutant VP1 (83K) protein of foot and mouth disease (FMDV) subverts MAVS signaling by disrupting the interaction between MAVS and TRAF3^101^. Moreover, the NS1 and N proteins of respiratory syncytial virus attenuate the production of type I IFNs during infection by inhibiting the MAVS/RIG-I interaction and by localizing MAVS in inclusion bodies, respectively^102,103^. The human metapneumovirus (hMPV) M2-2 protein prevents recruitment of the MAVS downstream adaptors TRAF3, TRAF5, and TRAF6^104^. Interestingly, a recent study showed that the M protein of SARS-CoV-2 impairs MAVS aggregation and the recruitment of downstream TRAF3, TBK1, and IRF3^105^, while another study reported that SARS-CoV-2 M2 inhibits RIG-I/MAVS/TRAF3 and TBK-1 complex formation and subsequent nuclear translocation of IRF3^26^. Viral proteins known to interact with or affect MAVS are listed in Table 4.

## Regulation of TRAF3 by host factors and viral proteins

TRAF3 (also called Amn, CAP-1, CD40bp, CRAF1, LAP1, or T-BAM) is one of the most enigmatic, ubiquitously expressed members of the TRAF family. The protein contains 568 amino acids (64.295 kDa) and a typical C3HC4 RING finger domain upstream of five zinc fingers, an isoleucine zipper, and a TRAF3 domain in the C-terminal region. The TRAF domain is critical for binding to the cytoplasmic domain of tumor necrosis factor receptor (TNFR) family members and intracellular signaling mediators and for the formation of homo- or heterodimers^106–109^. TRAF3 forms a stable complex with MAVS, which recruits kinases and IRF3 to itself, ultimately leading to IRF3 activation and nuclear translocation^110^.

The E3 ubiquitin ligases DEAD-box helicase 3 (DDX3)^110^, cIAP1, cIAP2^111^, galectin 3 binding protein (LGALS3BP)^112^, TRIM24^113^, and TRIM35^114^ trigger K63-linked polyubiquitination of TRAF3. This modification of TRAF3 enables its association with MAVS and TBK1, which activates downstream antiviral signaling. Moreover, the E3 ubiquitin ligase RING finger protein 166 transfers ubiquitin to TRAF3 upon RNA virus infection, thereby activating IFN-β production^115^. The serine-threonine kinase CK1ɛ interacts with TRAF3 and phosphorylates it on Ser349, which promotes Lys63 (K63)-linked ubiquitination of TRAF3 and subsequent recruitment of the kinase TBK1 to TRAF3^116^. Osteopontin (OPN) interacts with TRAF3 to inhibit Triad3A-mediated K48-linked polyubiquitination and degradation of TRAF3^117^. Downstream of kinase 3 (DOK3) interacts with TRAF3 through its tyrosine-rich CTD to induce TRAF3/TBK1 complex formation^118^, whereas the interaction between TRAF3 and the GTPase-trafficking protein RAB1B facilitates the formation of the TRAF3/MAVS complex^119^. As mentioned above, K63-linked polyubiquitination plays a critical role in activating TRAF3. Therefore, the deubiquitinases MYSM1^120^, DUBA^121^, USP19^122^, OTUB1, OTUB2^123^, UCHL1^124^, and FOSL1^125^ remove ubiquitin chains from TRAF3 to negatively regulate its function. In addition, scavenger receptor A (SRA) and HSCARG^126^ negatively regulate the stability of the TRAF3 protein by promoting recruitment of OTUB1 to TRAF3^127^. K48-linked polyubiquitination and degradation of TRAF3 mediated by estrogen receptor-alpha (ERα)^128^, WD repeat domain (WDR) 82^129^, Parkin^130^, and Triad3A^131^ is another mechanism that downregulates IFN production via targeting of TRAF3. Linear-ubiquitinated NEMO associates with TRAF3 and disrupts the MAVS-TRAF3 complex, thereby inhibiting IFN activation^132^.

Since K63-linked polyubiquitination plays an important role in TRAF3-mediated signaling, it comes as no surprise to see that viruses encode proteins that inhibit TRAF3 ubiquitination to overcome host innate responses. The leader proteinase (Lpro) of FMDV^133^ and the ubiquitin-specific protease (UL36) of herpes simplex virus 1 (HSV-1)^134^ act as viral deubiquitinases that mediate TRAF3 deubiquitination, leading to downregulation of TRAF3 signaling. The nonstructural protein 2A protease (2Apro) of human enterovirus D68 (EV-D68) cleaves TRAF3 at G462^135^. The M protein of SARS-CoV forms a complex with TRAF3, TANK, and the TBK1/IKKε complex to inhibit TBK1/IKKε-dependent activation of the IRF3/IRF7 transcription factors^27^.

## Regulation of NEMO by host factors and viral proteins

NF-κB essential modulator (NEMO or IKKγ), which contains 419 aa, is the integral regulatory scaffolding protein of the canonical IKK complex located at the center of both the NF-κB and type I IFN signaling cascades^136^. The IKK complex comprises two kinases, IKKα and IKKβ, and a regulatory subunit, NEMO^137^. For appropriate assembly of the IKK complex, NEMO contains two coiled-coil domains (CC1 and CC2) at its N-terminus upstream of a leucine zipper and a C-terminal zinc finger (ZF) domain. In response to RLR signaling, ubiquitinated TBK1 recruits the adaptor protein NEMO via the ubiquitin binding domain. Assembly of the NEMO/TBK1 complex on MAVS activates the TBK1 kinase and phosphorylation of IRF3^138^. As NEMO plays a critical role in regulating RLR-mediated IFN signaling, several positive and negative host regulatory factors (as well as viral proteins) play roles in regulating NEMO protein function. TRIM23-mediated K27-linked polyubiquitination of NEMO is crucial for virus-induced IRF3-mediated activation of RLR signaling. TRIM23-mediated ubiquitin conjugation occurs when NEMO K165, K309, K325, K326, and K344 are ectopically expressed^139^. Moreover, K48-linked polyubiquitination of NEMO mediated by the E3 ubiquitin ligases MARCH2 and TRIM29 leads to its proteasomal-dependent degradation^140,141^. RUN domain Beclin-1-interacting cysteine-rich-containing (Rubicon) interacts with NEMO and removes conjugated ubiquitin moieties from NEMO, thereby inhibiting its activation and subsequent signal transduction upon viral infection^142^. Additionally, progranulin (PGRN) is expressed during influenza virus infection; PGRN interacts directly with NEMO and recruits A20 (also called TNFAIP3), which removes K63-linked polyubiquitin chains from K264 of NEMO, resulting in impaired activation of downstream signaling^143^.

Viruses can escape antiviral immune responses by promoting cleavage or degradation of NEMO. Many viruses encode proteases that cleave NEMO independent of proteasomal degradation or apoptosis to inhibit RLR signaling. For example, 3C^90^ of FMDV specifically targets NEMO at Gln383, cleaving the C-terminal ZF domain from the protein and impairing the ability of NEMO to activate downstream IFN production^144^. Additionally, the HAV 3C protease (3Cpro) cleaves NEMO at Q304, thereby abolishing its signaling adaptor function and abrogating the induction of IFN-β synthesis^145^. NSP4, a viral 3C-like serine protease of PRRSV, cleaves NEMO at E166, E171, and E349–S350, while NSP4 of equine arteritis virus, which is similar to NSP4 of PRRSV, cleaves NEMO at E166, E171, Q205, and E349 to inhibit downstream signaling and maintain viral infection^146,147^. NSP5 of feline infectious peritonitis virus and NSP5 encoded by porcine epidemic diarrhea virus (PEDV) cleave NEMO at Q132, Q205, Q231, and Q231, resulting in downregulation of immune signaling^148,149^. Similarly, NSP5 of porcine deltacoronavirus (PDCoV) cleaves NEMO at Q231 to impair the ability of NEMO to activate the IFN response and downstream signaling^150^. Furthermore, ORF9b of SARS-CoV-2 disrupts K63-linked polyubiquitination of NEMO^151^, thereby downregulating IFN production during SARS-CoV-2 infection.

## Regulation of TBK1 by host factors

TRAF family member-associated NF-κB activator (TANK)-binding kinase 1 (TBK1, also called NAK or T2K) is one of two noncanonical IKKs implicated in regulating the activation of IRF3/IRF7 and the NF-κB signaling pathway. TBK1 is a 729 aa protein (84 kDa) containing an N-terminal kinase domain (KD), a ubiquitin-like domain (ULD), and two C-terminal coiled-coil domains^152^. The ULD acts as a regulatory domain by binding to the functional domains of TBK1 as well as to substrates such as IRF3/IRF7, thereby enabling the KD to phosphorylate target substrate proteins. Furthermore, the structure of TBK1 is similar to that of the noncanonical kinase IKKε; indeed, both kinases always work together. Cellular expression of TBK1 is ubiquitous; thus, it plays an indispensable role in antiviral innate immunity. Upon infection with RNA viruses, TBK1 is activated by the upstream protein MAVS, and activated TBK1 recruits IRF3 and IRF7; these proteins undergo TBK1-mediated C-terminal phosphorylation to trigger their dimerization and nuclear translocation, an event followed by induction of IFN secretion^153^.

As a vital kinase that regulates the activation of IRF3/IRF7 and the subsequent expression of IFN, the function of TBK1 must be regulated to maintain immune homeostasis and suppress viral replication. Therefore, several regulatory factors target TBK1 to control its function, while viruses have evolved mechanisms to disable it. Moreover, TRAF family E3 ubiquitin ligase-mediated K63-linked polyubiquitination of intact dimerized TBK1 at Lys30 and Lys401^154^ results in transautophosphorylation on Ser172, which marks TBK1 for phosphorylation-mediated activation^155^. Glycogen synthase kinase 3β (GSK3β) facilitates the aforementioned autophosphorylation of TBK1 at Ser172^156^. TRIM9 short isoform (TRIM9s) facilitates the recruitment of GSK3β to TBK1 upon viral infection^157^, and Raf kinase inhibitory protein serves as a positive regulator^158^; both of these proteins promote autophosphorylation of TBK1. Moreover, ubiquilin 2 (UBQLN2) promotes the stability and facilitates the phosphorylation of TBK1^159^, and Tyr179 (Y179) phosphorylation (catalyzed by the tyrosine kinase Src) is essential for the initiation of TBK1 autophosphorylation^160^. Ubiquitination also plays a critical role in the activation of TBK1. Mindbomb E3 ubiquitin-protein ligase 1 (MIB1) and MIB2^161^, ring finger protein 128 (RNF128)^162^, and neuregulin receptor degradation protein 1 (Nrdp1/RNF41)^163^ activate TBK1 by promoting its K63-linked ubiquitination. The deubiquitinase complex comprising ubiquitin-specific peptidase 1 (USP1) and USP1-associated factor 1 (UAF1), binds to TBK1 to remove K48-linked polyubiquitination and reverse the degradation process^164^. The DNA methyltransferase Dnmt3a maintains high expression of the histone deacetylase HDAC9, which maintains deacetylation of TBK1 and increases its kinase activity^165^, whereas HDAC3 positively regulates TBK1 in the same manner as HDAC9^166^. Moreover, butyrophilin 3A1 (BTN3A1) interacts with TBK1 to facilitate its dynein-dependent transport to the perinuclear region to promote its association with IRF3 after viral infection^167^. IFN-induced protein with tetratricopeptide repeats 3 (IFIT3) mediates the bridging of TBK1 to MAVS on mitochondria^168^. Additionally, the E3 ubiquitin ligase TRIM26 bridges the interaction between NEMO and TBK1, which facilitates immune activation upon viral infection^169^. Moreover, the homeobox protein MSX1 and docking protein 3 (DOK3) positively regulate TBK1 function to facilitate complex formation, and PLA1A upregulates TBK1 recruitment to mitochondria via modulation of mitochondrial morphology^118,170,171^.

In contrast, several TBK1-regulating proteins negatively impact TBK1. K48-linked polyubiquitination of TBK1 induced by E3 ubiquitin ligases such as SOCS box-containing 8 (ASB8)^172^, TRAF-interacting protein^173^, dual-specificity tyrosine phosphorylation-regulated kinase 2 (DYRK2)^174^, and THO complex subunit 7 homolog (THOC7)^175^ triggers proteasomal degradation of TBK1 and ultimately terminates immune activation. Interestingly, USP38 permits K48-linked ubiquitination and subsequent degradation of TBK1 by specifically removing K33-linked ubiquitin chains from the same lysine site on TBK1^176^. Additionally, Siglec1 recruits TRIM27 and NLRP4 recruits DTX4 to trigger K48-linked polyubiquitination of TBK1^177,178^. As noted above, K63-linked polyubiquitination plays a crucial role in activating TBK1. Therefore, any protein that disrupts the ubiquitin chain can be considered a negative regulator. For example, the deubiquitinating enzyme cylindromatosis (CYLD) removes K63-linked polyubiquitin moieties from TBK1^34^, and the A20 regulatory complex (comprising the ubiquitin-editing enzyme A20, Tax1-binding protein 1 (TAX1BP1, also called T6BP or TXBP151)^179^, and ubiquitin-specific protease (USP) 2b (USP2b)^180^ antagonize K63-linked polyubiquitination of TBK1. Moreover, UBE2S recruits USP15 to TBK1, thereby removing K63-linked polyubiquitin chains^181^. The Src family kinases Lck, Hck, and Fgr phosphorylate TBK1 directly at Tyr354/394 to prevent its dimerization and activation^182^. The ADP-ribosylase TIPARP interacts with TBK1 to suppress its activity via ADP-ribosylation^183^. The phosphatase Cdc25A dephosphorylates TBK1 at its activation site (S172) upon viral infection^184^. Moreover, upon infection with RNA viruses, protein phosphatase 1B (PPM1B)^185^, Cdc25A^184^, and protein phosphatase 4 (PP4)^186^ dephosphorylate Ser172 of TBK1 to prevent continuous activation of TBK1. Preventing protein–protein interactions is another method of inhibiting TBK1-driven immune activation. NOD-like receptors (e.g., NLRP2)^187^ and estrogen-related receptor α (ERRα)^188^ inhibit the interaction between TBK1 and IRF3, while MIP-T3^189^ prevents the formation of the TRAF3/TBK1 complex. Additionally, ISG56 disrupts the interaction between MITA and VISA or TBK1, while INKIT interacts with TBK1 to impair the recruitment and phosphorylation of IRF3^190,191^.

## Regulation of TBK1 by RNA viral proteins

TBK1 is targeted by viruses to modulate innate immune activation and ensure viral survival and persistent replication. SARS-CoV-2 virus NSP13^192^, Heartland virus (HRTV) NS^193^, and dengue virus (DENV) serotype 4 (DENV4) NS^194^ proteins interact directly with TBK1 to prevent its autophosphorylation. Papain-like protease domain 2 (PLP2) of mouse hepatitis virus A59 (MHV-A59)^195^ and the short form leader proteinase (Lpro) Lbpro of FMDV^133^ cleave ubiquitin chains from TBK1 and inactivate its kinase activity. The Us11 protein of HSV-1 interacts with Hsp90, which competes with TBK1 to disrupt the formation of the TBK1/Hsp90 complex. Us11 subsequently mediates TBK1 destabilization via a proteasome-dependent pathway^196^. Severe fever with thrombocytopenia syndrome bunyavirus (SFTSV) escapes the host immune system by inducing the formation of cytoplasmic inclusion bodies with the help of NS proteins^197,198^, whereas the NS protein of SFTSV impairs the autophosphorylation of TBK via a direct interaction^199^. Moreover, the N protein of PEDV^200^ and the NS protein of HRTV^201^ inhibit the TBK1/IRF3 interaction by targeting TBK1 directly, while the NS5 protein of Zika virus antagonizes IFN production by blocking TBK1 activation^202^. A recent study demonstrated that NSP13 of SARS-CoV-2 interacts directly with the MAVS binding domain of TBK1 and disrupts the TBK1-MAVS interaction^203^. Membrane-anchored PLpro domain (PLpro-TM) of SARS-CoV inhibits STING/TBK1/IKKε-mediated activation of type I IFNs by disrupting the phosphorylation and dimerization of IRF3^204^. FLIPs (MC159 and MC160) encoded by molluscum contagiosum virus inhibit TBK1 phosphorylation and activation; however, MC159 interacts directly with TBK1, whereas MC160 does not^205^. Grass carp reovirus (GCRV) inhibits TBK1 activation by removing K63-linked ubiquitination from TBK1 and promoting its K48-linked ubiquitination^24^.

## Regulation of IKKε by host factors and viral proteins

IKKε (originally called IKKi) is a noncanonical member of the IκB kinase family that has been studied extensively due to its ability to promote type I IFN responses. IKKε is a 716 aa protein comprising a KD, a ULD, and a scaffold dimerization domain. The KD of IKKε shares 49% identity and 65% similarity with that of TBK1^206^. Activation of TBK1 and IKKε promotes phosphorylation and nuclear translocation of IRF3 and 7, leading to transcriptional upregulation of type I IFNs during the induction of the innate immune response^207^. During the innate immune response, TBK1 and IKKε exhibit functional redundancy, although TBK1 appears to be more important than IKKε. The IKK subunit NEMO promotes activation of TBK1 and IKKε downstream of cytoplasmic DNA signaling, whereby ubiquitinated NEMO recruits IKKβ to facilitate activation of TBK1 or IKKε.

Biochemical analysis has revealed that the interaction between sphingosine 1-phosphate (S1P) lyase and IKKε leads to IKKε-driven activation of IFN signaling^208^. Viral infection triggers an interaction between DDX3 and IKKε. Expression of DDX3 amplifies TBK1/IKKε-mediated induction of the IFN-β promoter^209^. DExD/H-box RNA helicase 19 (DDX19) recruits Lamtor2 to form the TBK1/IKKε/Lamtor2/DDX19/IRF3 complex, which suppresses IFN production by promoting degradation of TBK1 and IKKε^210^. Fascin1, an actin-bundling protein, interacts with IKKε to suppress the RIG-I-mediated signaling cascade in colon cancer cells^211^.

To date, few studies have been conducted on viral proteins that interfere with the signaling mechanisms of IKKε. NS2B/3 of DENV interacts directly with IKKε; computational analysis revealed that via this interaction, NS2B/3 masks the KD of IKKε and potentially affects its functionality, thereby impairing the phosphorylation and nuclear translocation of IRF3^212^. Interestingly, NS2 of HCV interacts physically with the IKKε/TBK1 kinase complex, thereby inhibiting IRF3 phosphorylation^213^. Moreover, the VP35 protein of Ebola virus (EBOV) interacts with IKKε and TBK1 during the early phase of viral infection; this physical interaction with IKKε further prevents the interaction of IKKε with IRF3, IRF7, and MAVS^214^. Similarly, lymphocytic choriomeningitis virus (LCMV) NP binds to the KD of IKKε to block its autocatalytic activity and its ability to phosphorylate IRF3^215^. Additionally, ORF8b of Middle East respiratory syndrome coronavirus (MERS-CoV) inhibits HSP-70-dependent IKKε activation, while NS2 of HCV inhibits IKKε-dependent phosphorylation of IRF3^213,216^.

## Regulation of IRF3 by host factors

IRF3 (also called IIAE7) is a master transcription factor responsible for the induction of innate antiviral immunity. It is a 427 aa (47.219 kDa) protein that is expressed ubiquitously in tissues. IRF3 contains an N-terminal DNA binding domain (DBD) and a C-terminal transactivation domain. After considerable research, TBK1 and IKKε were identified as the kinases responsible for IRF3 phosphorylation at its C-terminus, which facilitates the formation of dimers that are then transported to the nucleus^136,217^ to form a complex with coactivators of the p300/CBP family and initiate the transcription of target genes, including the gene encoding IFN-β^218,219^. IRF3 contains an active nuclear localization signal that is recognized by importin-α receptors and results in its transport into the nucleus^219,220^.

Because IRF3 is crucial for RLR-mediated antiviral immune activation, it is not surprising that IRF3 function is both positively and negatively regulated by host proteins or that viruses have evolved mechanisms to abolish protein expression. The long noncoding RNA (lncRNA) lncLrrc55-AS recruits methylesterase 1 (PME-1) to promote the interaction between PME-1 and the phosphatase PP2A, an inhibitor of IRF3 phosphorylation^221^. Similarly, IRF1 interacts with IRF3 to augment the activation of IRF3 by blocking the interaction between IRF3 and PP2A^222^. Heat shock protein family D (Hsp60) member 1 facilitates the phosphorylation and dimerization of IRF3 and increases IFN-β induction induced by SeV infection^61^. The lysine methyltransferase nuclear receptor-binding SET domain 3 (NSD3) binds directly to the IRF3 C-terminal region through its PWWP1 domain and methylates IRF3 at K366. Monomethylation maintains IRF3 phosphorylation by promoting the dissociation of IRF3 from the protein phosphatase PP1cc, thereby promoting the production of type I IFNs^223^. The deubiquitinating enzyme USP22 deubiquitinates and stabilizes KPNA2 after viral infection, thereby facilitating efficient nuclear translocation of IRF3^224^.

Regarding the negative regulation of IRF3-mediated signaling, the E3 ubiquitin ligase interacting protein peptidyl-prolyl cis/trans isomerase, NIMA-interacting 1^225^, and RBCC protein interacting with PKC1 (RBCK1)^226^, Ro52/TRIM21^227^, the HECT domain ubiquitin^228^ E3 ligase RAUL^229^, and TRIM26^230^ catalyze the K48-linked polyubiquitination and subsequent proteasomal degradation of IRF3. Moreover, OTUD1 removes viral infection-induced K6-linked ubiquitin moieties from IRF3, resulting in dissociation of IRF3 from the promoter region of its target genes without affecting its protein stability, dimerization, or nuclear translocation^231^. IFN-induced transmembrane protein 3 (IFITM3) associates with IRF3 and regulates the homeostasis of IRF3 by mediating its autophagic degradation^232^. Phosphorylation of IRF3 is the key modification that leads to its activation. Therefore, dephosphorylation of IRF3 via phosphatases such as MAPK phosphatase 5 (MKP5)^233^ and the serine/threonine phosphatase PP2A^234^ inactivates IRF3. However, Mst1 associates with IRF3 and phosphorylates IRF3 directly at Thr75 and Thr253, which prevents IRF3 homodimerization, reduces its ability to occupy chromatin, and dampens IRF3-mediated transcriptional responses^235^. Interestingly, the F-box protein FBXO17 decreases IRF3 dimerization and nuclear translocation by recruiting protein phosphatase 2A (PP2A), resulting in dephosphorylation of IRF3^236^; research suggests that the DDX5 protein facilitates this process during viral infection^237^. HDAC4 inhibits TBK1- and IKKε-mediated phosphorylation of IRF3 at Ser386 and Ser396^238^. Sentrin/SUMO-specific protease 2 (SENP2) causes IRF3 deSUMOylation, K48-linked ubiquitination, and degradation^239^. DEAD-box polypeptide 56 (DDX56) suppresses the nuclear translocation of IRF3 by disrupting the interaction between IRF3 and the nuclear translocation supporter IOP5^240^. Rubicon specifically interacts with the IRF association domain (IAD) of IRF3, which prevents dimerization of IRF3^241^. Human argonaute 2 (AGO2) blocks the association of IRF3 with CBP; however, this interaction does not affect the phosphorylation, nuclear translocation, or DNA binding of IRF3^242^.

## Regulation of IRF3 by RNA viral proteins

Due to genomic constraints, the immunomodulatory efforts of most viruses focus on host targets that are key players in the antiviral response. It is not surprising, therefore, that IRF3 is one of these targets. The NS1 proteins of influenza A virus (IAV)^243^ and porcine hemagglutinating encephalomyelitis virus (PHEV)^244^, the phosphoprotein (P) of rabies virus (RABV)^245^, the PLpro protein (with deubiquitination activity) of SARS-CoV-2, the NSP1β protein of PRRSV^228^, the N protein of Peste des petits ruminants virus (PPRV)^247^, and the NSP15 protein of PEDV^248^ inhibit activation of IRF3 to downregulate nuclear translocation. A recent study reported that open reading frame 6 (ORF6) of SARS-CoV-2 binds to the importin karyopherin α 2 (KPNA2), thereby inhibiting the nuclear translocation of IRF3^192^; in addition, the ORF6, NSP12, and NSP5 proteins inhibit the nuclear translocation of IRF3 to prevent IFN production^249^^,^^250,251^, while the NSP3/papain-like protease cleaves IRF3 to subvert IFN production^252^. Moreover, NS5 of Japanese encephalitis virus (JEV) interacts with the nuclear transport proteins KPNA2, KPNA3, and KPNA4, which competitively block the interactions between KPNA3 and KPNA4 and one of their cargo molecules, IRF3^253^. JEV downregulates IRF3 phosphorylation and nuclear translocation, an effect that became more pronounced when the molar ratio of sfRNA to genomic RNA was increased^254^. The V protein of Sendai virus (SeV) inhibits IRF3 translocation to the nucleus^255^, and the 3C_pro_ protein of SVV degrades IRF3 via its protease activity^256^. The N_pro_ protein of classical swine fever virus (CSFV)^257^ and the NSP1 protein of RV^258^ trigger proteasomal degradation of IRF3. FMDV 3A interacts with DDX56 to inhibit type I IFN production by reducing the phosphorylation of IRF3^259^. Hantavirus^260^ oncoprotein Tax of human T-cell leukemia virus type 1 (HTLV-1)^261^, the NS protein of DENV^194^, and the M protein of MERS-CoV^262^ downregulate IRF3 phosphorylation. Moreover, two reports revealed that the ML protein of Thogoto virus (THOV) and the NSP1 protein of RV block the dimerization and subsequent nuclear translocation of IRF3^263,264^.

## Regulation of IRF7 by host factors and RNA viral proteins

IRF7 is a 503 aa (55 kDa) protein containing an N-terminal DBD, an IAD, a nuclear export sequence, an autoinhibitory domain, and a signal response domain composed of key serine residues^217,265^. Unlike IRF3, IRF7 is not expressed ubiquitously in cells; instead, its expression is induced upon pathogen infection or stimulation. However, it is a master regulator of type I IFN gene expression and IFN-dependent innate immune responses^266^. IKKε and TBK1 are the major kinases responsible for IRF7 phosphorylation and activation^267^. Nuclear translocation and accumulation of IRF7 trigger the induction of IFN-β and IFN-α expression^268^. K63-linked polyubiquitination of IRF7 on lysines 444, 446, and 452, a process that is important for its activation prior to its phosphorylation and nuclear translocation, is triggered by TRAF6^269^. Research has shown that the regulation of IRF7 activity by several negative regulators maintains immune homeostasis. N-Myc and STAT interactor (Nmi) promote K48-linked ubiquitination of IRF7 and its subsequent proteasome-dependent degradation^270^, whereas Ro52/TRIM21 mediates its ubiquitination-promoted degradation upon upstream signaling activation^271^. TRIM28 interacts with the SUMO E2 enzymes to increase the SUMOylation of IRF7. TRIM28-mediated SUMOylation of IRF7 increases during viral infection, resulting in transcriptional repression^272^. The N-terminal deubiquitinase^273^ domain of the enzyme A20 interacts physically with IRF7 to reduce its K63-linked ubiquitination and negatively regulate transcriptional function^274^. Moreover, physical interactions between IRF7 and the IFN-inducible p200 family protein IFI204^275^, activating transcription factor 4 (ATF4), and HSP70^276,277^ downregulate IRF7 activity, leading to downregulation of innate immune activation. Different RNA viral proteins inhibit IRF7. VP35 of EBOV increases PIAS1-mediated SUMOylation of IRF7, thereby repressing IFN transcription^278^. In addition, HCV infection impairs the nuclear translocation of IRF-7^279^. The Zn-binding domain of the CSFV N_pro_ protein interacts directly with IRF7 to subvert its function^280^. In particular, 3C_pro_ of SVV was found to reduce IRF7 protein expression and phosphorylation in PK-15 cells^256^.

## DNA virus-induced signal transduction and immune evasion mechanisms

Upon infection with DNA viruses, viral DNA is released into the host cell cytoplasm prior to viral protein synthesis. Cytosolic viral DNA is recognized mainly by cyclic GMP-AMP (cGAMP) synthase (cGAS), which contains a nucleotidyltransferase (NTase) domain. After DNA binding, cGAS synthesizes a second messenger molecule, cyclic GMP-AMP (cGAMP). This cGAMP isomer, called 2’,3’-cGAMP, functions as a second messenger that binds to the ER membrane adaptor STING^281–283^ to induce a conformational change that presumably results in activation of STING. STING then traffics from the ER to the ER-Golgi intermediate compartment and then to the Golgi apparatus^284,285^. During this process, the carboxyl terminus of STING recruits and activates the kinase TBK1, which in turn phosphorylates the transcription factor IRF3. Phosphorylated IRF3 dimerizes and then enters the nucleus, ultimately leading to the induction of type 1 IFN genes and other antiviral genes^286^. Although other proteins, such as IFI16, DDX41, and MRE11, also mediate DNA-induced IFN-β production in a STING-dependent manner, only cGAS, which enzymatically generates cGAMP as a second messenger that activates STING, provides a clear molecular mechanism for DNA-stimulated IFN-β production^287^. However, DNA viruses exploit strategies to evade innate immune responses. Below, we describe the activation and regulation of these mechanisms, along with the immunomodulatory mechanisms by which viruses evade them.

## Regulation of 2′,3′-cGAMP by host factors and viral proteins

Upon DNA recognition, cGAS generates the second messenger 2′,3′-cyclic GMP-AMP (2′,3′-cGAMP) by using ATP and GTP^284,288^. Unlike the secondary messengers in classical bacterial signaling (c-di-GMP and c-di-AMP), 2′,3′-cGAMP contains mixed phosphodiester bonds (G(2′,5′)pA and A(3′,5′)pG)^282,289^. The intermediate product, called 5′-pppG(2′,5′)pA, is generated by cGAS prior to synthesis of cyclic 2′,3′-cGAMP^42^. Next, 2′,3′-cGAMP interacts with STING to activate downstream signaling, resulting in strong induction of IFNs, which confer antiviral efficacy^288^. To date, few studies have examined host factors and viral proteins that regulate 2′,3′-cGAMP function during innate immune activation. A recent study of HSV-1 infection showed that Leucine-rich repeat-containing protein (LRRC) LRRC8A/LRRC8E-containing volume-regulated anion channels transport cGAMP across the plasma membrane to initiate effective antiviral innate immunity^290^. In contrast, 2′,3′-cGAMP is hydrolyzed predominantly by ectonucleotide pyrophosphatase/phosphodiesterase (ENPP1), thereby preventing STING activation. In general, viruses have evolved mechanisms to antagonize host innate immune activation^291,292^. However, the antiviral second messenger 2′,3′-cGAMP can be packaged into viral particles, including those of poxviruses, herpesviruses, and retroviruses, thereby enabling its transfer to newly infected cells, where it activates the immune response. Once 2′,3′-cGAMP-carrying virions infect neighboring cells, they activate a STING-dependent antiviral program^293,294^. Moreover, the poxvirus immune nuclease (poxin) family, a family of 2′,3′-cGAMP-degrading enzymes, has been identified. Vaccinia virus poxin degrades 2′,3′-cGAMP through metal-independent cleavage of the 3′-5′ bond, thereby converting 2′,3′-cGAMP into linear Gp[2′-5′]Ap[3′]. Furthermore, the same study revealed that deletion of the poxin gene (B2R) attenuates vaccinia virus replication in vivo, thereby restricting STING-dependent signaling^295^.

## Regulation of STING by host factors

STING, also called MITA, ERIS, TMEM173, or MPYS, is an ER membrane signaling^283^ protein of 379 aa; it harbors a predicted TM portion (aa residues 1–173) at the N-terminus, which regulates its cellular localization and homodimerization, since the TM domains cross the ER membrane. It also harbors an intracellular soluble portion (aa residues 174–379) in the CTD, which functions to dock downstream molecules such as TBK1/IKKε and IRF3/IRF7^296,297^. To initiate signaling, the native ligand cGAMP binds to the V-shaped hydrophilic pocket in the STING dimer. The resulting conformational change exposes the hidden CTT of STING to TBK1 and IRF3^298,299^. Due to this conformational change, STING is transported from the ER to the ER-Golgi intermediate compartment and then to the Golgi apparatus and perinuclear region^300^.

Since STING is essential for innate immune responses to cytosolic nucleic acids, its activity is tightly regulated to maintain immune homeostasis while enabling timely activation of downstream signaling to fight against viral infections. Several PTMs are involved in regulating STING function. Among them, K63-linked polyubiquitination plays a critical activating role. Mitochondrial E3 ubiquitin protein ligase 1 (MUL1) catalyzes K63-linked polyubiquitination of STING at K224 to transport TBK1 to IRF3. The ubiquitination-deficient STING mutant K224R fails to translocate to perinuclear puncta in response to a stimulus, suggesting that K63-linked polyubiquitination of STING at K224 is essential for STING trafficking^301^. The E3 ubiquitin ligases TRAF6^302^, ubiquitin regulatory X domain-containing protein-3B (UBXN3B)^303^, and RNF115^59^ also conjugate K63-linked polyubiquitin chains to STING, thereby strengthening its interaction with IRF3 and TBK1. The E3 ubiquitin ligase complex AMFR and insulin-induced gene 1 (INSIG1) catalyze K27-linked polyubiquitination of STING. This modification acts as an anchoring platform for recruitment of TBK1, thereby facilitating its translocation to perinuclear microsomes^304^. K48-linked polyubiquitination is one of the main negative regulatory mechanisms of cellular STING protein expression. Therefore, any factor that disrupts the K48-linked polyubiquitin chain may activate signal transduction. The DUBs USP20/USP18^305,306^, USP44^307^, CYLD^308^, OTUD5^309^, and iRhom2^310^ remove K48-linked polyubiquitin chains from STING and ultimately boost innate antiviral responses. Palmitoylation plays an important role in regulating protein transport, stability, and cellular localization in host cells. Palmitoylation of STING occurs after its trafficking to the Golgi apparatus; this PTM is essential for activation of STING. Moreover, the palmitoylation inhibitor 2-bromopalmitate (2-BP) impairs STING-mediated IFN induction^311,312^. Phosphorylation of STING by TBK1 at S366 promotes the recruitment and activation of IRF3^313^. Moreover, S358 of STING is also phosphorylated, although the kinase responsible is not known^314^. Interestingly, upon DNA virus infection, the tyrosine kinase CSK phosphorylates STING at Y240 and Y245, which is important for its activation^315^. The ER-associated proteins ZDHHC1 and transmembrane emp24 protein transport domain-containing 2 (TMED2) associate with STING and mediate its dimerization/aggregation; they also facilitate its trafficking^315,316^. SNX8 recruits the class III phosphatidylinositol 3-kinase protein VPS34 to STING, thereby facilitating the trafficking of STING from the ER to perinuclear microsomes^317^.

With respect to the negative regulation of STING, RNF5 impairs STING signaling by modifying it at K150 through K48-linked polyubiquitination, which promotes its degradation^318^. RNF90 and TRIM29 also promote K48-linked ubiquitination of STING and impair STING signaling^319,320^; however, the specific aa residue^1^ that is ubiquitinated is not defined. Moreover, TRIM30α negatively regulates the STING pathway via K48-linked ubiquitination of STING on K275^321^. In contrast, the DUB USP21 hydrolyzes K27/63-linked polyubiquitin chains^322^, USP49^323^ removes K63-linked polyubiquitin chains, and USP13^324^ removes K33-linked polyubiquitin chains on STING to negatively regulate STING-mediated signaling. PPM1A dephosphorylates STING at S358 and suppresses the formation of perinuclear puncta, thereby suppressing immune responses^314^. Phosphorylation of Y245 on STING is critical for STING activation. PTPN1 and PTPN2 dephosphorylate STING at Y245 and then promote its degradation via the 20 S proteasome^325^. Additionally, MRP^326^ and NLRX1^327^ interact with STING to downregulate its function, while RIG-I and IL-6 trigger proteasomal degradation of STING in human diploid cells upon dsDNA stimulation^328^. Autophagy-related gene 9a (Atg9a) colocalizes with STING to disrupt the binding of STING to TBK1^329^.

## Regulation of STING by viral proteins

STING plays a critical role in the host defense against infections with DNA viruses such as HSV-1, vaccinia virus (VVΔE3L), cytomegalovirus (CMV), and baculoviruses^330^. Therefore, viruses have evolved certain strategies to defeat host innate immunity by antagonizing STING signaling. For example, the ICP27^331^ protein of HSV is translocated to the cytoplasm during infection, where it interacts with STING and inhibits IRF3 activation. The HSV-1 γ34.5 protein downregulates STING trafficking from the ER to the Golgi by interacting with the N-terminus of STING^332^, while UL46 of HSV-1, one of the most abundant HSV tegument proteins, interacts with STING to prevent its activation^333^. The HSV-1 VP1-2 protein deubiquitinates STING and inhibits its downstream signaling^334^. The human T lymphotropic virus type 1 (HTLV-1) Tax protein also deubiquitinates STING to inhibit its downstream signaling^22^, while NS4B of HCV cleaves STING directly^335^, and vIRF1 of KSHV impairs the STING/TBK1 interaction^336^. Murine CMV (MCMV) encodes a product referred to as M152, which interacts with STING to suppress its activation^337^. The viral polymerase of HBV interferes with K63-linked polyubiquitination of STING via its reverse transcriptase domain^338^. The HCMV tegument protein UL82 negatively regulates STING signaling by interacting directly with STING. It then inhibits STING trafficking from the ER to perinuclear punctate structures^339^. The IE86 protein of HCMV facilitates proteasome-dependent degradation of STING to suppress the secretion of IFN-β1 and CXCL10^340,341^, and UL42 of HCMV impairs the translocation of STING from the ER to perinuclear punctate structures, which is required for STING activation^342^. Duck Tembusu virus (DTMUV) NS2B3 cleaves STING by interacting with aa residues 221–225; this method of STING cleavage is not strictly species-specific^343^.

## Regulation of TBK1 by DNA viral proteins

To complete their life cycles in the host, DNA viruses use numerous strategies to evade host immune signaling initiated by RLRs; they do this by targeting TBK1. The Us11 protein of HSV-1 associates with endogenous Hsp90 to disrupt the Hsp90/TBK1 complex, which blocks TBK1 activation. Furthermore, Us11 induces destabilization of TBK1 through a proteasome-dependent pathway that ultimately blocks phosphorylation of IRF 3^196^. In addition, the UL46 protein of HSV-1 interacts with the C-terminal region of TBK1 to inhibit the interaction of TBK1 and STING^333^, whereas the gamma(1)34.5 protein forms a complex with TBK1 and disrupts the TBK1/IRF3 interaction, thereby preventing downstream signaling^344^. ORF11 of murine gammaherpesvirus 68 (MHV-68) interacts directly with TBK1; in particular, it inhibits the TBK1/IRF3 interaction^345^. The C-terminus and the coiled-coil domain of feline panleukopenia virus (FPV) NS2 interact physically with TBK1, thereby preventing it from being recruited by STING; ultimately, this disrupts the phosphorylation of the downstream protein IRF3^346^.

## Regulation of IRF3 by DNA viral proteins

A number of DNA viral proteins inhibit IRF3 to suppress innate immune signaling. The VP24 protein of HSV-1 and the LANA2 (also called vIRF3) protein of Kaposi’s sarcoma-associated herpesvirus (KHSV) limit the induction of IFN-β by interacting with IRF3 to inhibit its dimerization and phosphorylation^29,347^. The ICP0 protein (bICP0) encoded by bovine herpesvirus 1 (BoHV-1) induces proteasomal degradation of IRF3 but not IRF7^348^. Varicella-zoster virus (VZV) is an important alpha herpesvirus that infects only humans. Several VZV viral proteins interfere with IRF3 activity. VZV viral immediate-early protein 62 (IE62) inhibits IRF3 phosphorylation at key serine residues but does not interfere with the IRF3/TBK1 interaction^349^. ORF47 interacts directly with IRF3, thereby inhibiting subsequent signal transduction, while ORF61 interacts directly with IRF3 and induces its ubiquitination and proteasomal degradation^350,351^. The nuclear early protein N2 of vaccinia virus inhibits the phosphorylation and nuclear translocation of IRF3^351^.

## Regulation of IRF7 by DNA viral proteins

Different viral proteins inhibit and activate IRF7. The interaction of the Epstein-Barr virus oncoprotein LMP1 with IRF7 catalyzes RIP-dependent K63-linked polyubiquitination and subsequent activation of IRF7^352^. The VP23 protein of Marek’s disease virus interacts with IRF7 and blocks its binding to TBK1, thereby inhibiting IRF7 phosphorylation and nuclear translocation, resulting in reduced IFN-β production^353^. The immediate-early nuclear transcription factor RTA encoded by KSHV and HHV8 acts as an ubiquitin E3 ligase to catalyze the polyubiquitination and proteasomal degradation of IRF7^354^. KSHV vIRF3 interacts specifically with either the DBD or the central IAD of IRF7, which inhibits the DNA binding activity of IRF7^355^. KSHV vIRF4 interacts specifically with IRF7, thereby inhibiting IRF7 dimerization and ultimately suppressing IRF7-mediated activation of type I IFNs^356^. LANA2 (also called vIRF3) of KSHV limits the induction of IFN-β by interacting with IRF7 and inhibiting its phosphorylation^29^.

## Conclusions

Over the past few decades, tremendous research progress has been made in identifying and characterizing two antiviral innate immunity pathways: the RLR-MAVS pathway for cytoplasmic RNA sensing and the cGAS-cGAMP-STING pathway for cytosolic DNA recognition. In this review, we summarize the current knowledge of the mechanisms that positively and negatively regulate PRR-mediated immune responses. We also discuss the molecules involved in the two abovementioned signaling pathways, which maintain immune homeostasis to achieve the most favorable outcome for the host. Finally, we explain how viral proteins adapt to escape host antiviral mechanisms to maintain active infection.

Due to advanced biomedical techniques such as fluorescence imaging, mass spectrometry, and nuclear magnetic resonance imaging, we now know much more about the molecular mechanisms and the host and viral factors that regulate signaling. Moreover, each new regulatory and molecular mechanism identified brings the inspiring possibility that we may identify and develop novel immunostimulatory agents, anti-inflammatory agents, vaccines, and antiviral agents that tilt the host-pathogen interaction in favor of the host. Despite tremendous advances in our knowledge regarding the functions and mechanisms of positive and negative regulatory molecules and of escape mechanisms used by viruses to evade innate immune signaling, several intriguing and important aspects regarding the regulation of RNA- and (especially) DNA-initiated signaling pathways and viral escape mechanisms remain elusive. These will be interesting topics for future investigations.
